# Strategies for overcoming tumour resistance to immunotherapy: harnessing the power of radiation therapy

**DOI:** 10.1093/bjr/tqae100

**Published:** 2024-06-04

**Authors:** Katiuska Passelli, David Repáraz, Remy Kinj, Fernanda G Herrera

**Affiliations:** Centre Hospitalier Universitaire Vaudoise, Service of Radiation Oncology, Department of Oncology, University of Lausanne, AGORA Center for Cancer Research, Swiss Cancer Center Leman, 1012-Lausanne, Switzerland; Centre Hospitalier Universitaire Vaudoise, Service of Radiation Oncology, Department of Oncology, University of Lausanne, AGORA Center for Cancer Research, Swiss Cancer Center Leman, 1012-Lausanne, Switzerland; Centre Hospitalier Universitaire Vaudoise, Service of Radiation Oncology, Department of Oncology, University of Lausanne, 1012-Lausanne, Switzerland; Centre Hospitalier Universitaire Vaudois, Service of Radiation Oncology and Service of Immuno-oncology, Department of Oncology, University of Lausanne, Ludwig Institute for Cancer Research, Agora Center for Cancer Research, Swiss Cancer Center Leman, 1012-Lausanne, Switzerland

**Keywords:** radiotherapy, radiation oncology, immunotherapy, low dose radiation, abscopal

## Abstract

Immune checkpoint inhibitors (ICI) have revolutionized cancer treatment; yet their efficacy remains variable across patients. This review delves into the intricate interplay of tumour characteristics contributing to resistance against ICI therapy and suggests that combining with radiotherapy holds promise. Radiation, known for its ability to trigger immunogenic cell death and foster an *in situ* vaccination effect, may counteract these resistance mechanisms, enhancing ICI response and patient outcomes. However, particularly when delivered at high-dose, it may trigger immunosuppressive mechanism and consequent side-effects. Notably, low-dose radiotherapy (LDRT), with its capacity for tumour reprogramming and reduced side effects, offers the potential for widespread application. Preclinical and clinical studies have shown encouraging results in this regard.

## Introduction

Immune checkpoint inhibitors (ICI) that target cytotoxic T-lymphocyte-associated protein 4 (CTLA-4) and programmed cell death protein 1 (PD-1) and its ligand (PD-L1) have significantly transformed the treatment of a variety of cancers, such as melanoma, lung and genitourinary cancers. However, ICI treatments are not available to more than half of cancer patients, and only half of those who are suitable will benefit.^1^ Numerous studies have been conducted in an attempt to uncover predictive biomarkers of response, which have so far been confined to PD-L1 expression,^2^ tumour mutational burden (TMB),^3^^,^^4^ tumour CD8^+^ T cell abundance, and DNA repair deficiencies.^5^ Moreover, the clonal expansion of T-cells in peripheral blood or tumour samples has been associated with long-term sustained responses to ICI therapy.^6^ Therefore, already existing immune cells may serve as predictors for ICI treatment responses; nevertheless, only a small fraction of tumours exhibit a significant infiltration of relevant T cells at the steady state. Although CD8^+^PD-1^high^ tumour-infiltrating lymphocytes (TILs) abundance is considered the pivotal factor for predicting ICI therapy response,^7^^,^^8^ some studies indicate that a subset of these T cells, known as precursor exhausted CD8^+^ T cells (also known as stem-like CD8^+^ T cells), which follow a canonical CD8 activation observable in antiviral responses, downregulate the transcription factor T cell factor-1 (encoded by *Tcf7* gene) and coexpress a plethora of coinhibitory receptors along with costimulatory and chemokine receptors, indicative of both activation and dysfunction, while proving the opportunity for re-invigoration.^9–12^ As a result, the ratio of exhausted T cell reinvigoration to tumour burden is proposed as a predictor of clinical efficacy.^13^ However, reinvigoration of CD8^+^ T cells cannot always be achieved by anti-PD1 administration.

In addition, inflamed tumours may also be subject to suppression by regulatory T cells (Tregs),^14^^,^^15^ myeloid derived suppressor cells (MDSCs),^16^ and tumour-associated macrophages (TAMs),^17^^,^^18^ as well as a plethora of soluble factors both produced by the aforementioned cells and tumour stroma, among which we can mention interleukin(IL)-10,^19^ prostaglandins,^20^ and transforming growth factor-β (TGF-β),^21^ which may hamper an effective immune response. In other instances, T cells are excluded from the tumour islets due to vascular barriers such as upregulation of FasL on endothelial cells, which selectively kills T cells while leaving Tregs unaffected,^22^ or the downregulation of endothelial adhesion molecules intercellular adhesion molecules (ICAM) and vascular cell adhesion molecule-1 (VCAM) which are important for T cell extravasation.^23^ Similarly, T cells require the support of conventional type 1 dendritic cells 1 (cDC1) in the tumour microenvironment (TME), a highly specialized antigen presenting cell (APC) subtype capable of presenting tumour antigens and producing the necessary chemokines for enhanced T cell migration (ie, CXCL9/CXCL10).^24–26^ Moreover, other immune cells, such as tumour associated neutrophils (TANs) might block T cell infiltration through neutrophil extracellular traps (NET), which can protect the tumour from natural killer cells (NKs) and cytotoxic T cell attack.^27^ In pancreatic ductal adenocarcinoma (PDAC), cancer cell-autonomous CXCL1 attracts TANs which subsequently exclude T cells from the TME.^28^

Attempts are being made to increase ICI responses by combinatorial approaches that promote T cell trafficking into the tumour, reinvigorate adaptive immune responses, or reduce immunosuppressive factors in the TME.^29^ In line with this, radiation therapy (RT) can have significant immunomodulatory effects, such as exposing tumour antigens via *in situ* vaccination^30–32^ and reprogramming the TME through T cell attracting cytokines and chemokines,^33^ normalization of the tumour vasculature,^34^ and activation of DCs.^35^^,^^36^ In essence, RT can promote T cell homing, migration into the tumour bed, tumour cell recognition, and effector function. It is also now well recognized that RT can increase the pool of precursor exhausted T cells.^37^^,^^38^ There is also increasing evidence of its ability to enhance systemic antitumour immunity, allowing for the control of distal metastases in exceptional circumstances, the so-called abscopal effect,^39^ and to synergize with immunotherapy in preclinical investigations^40^^,^^41^ and in patients.^39^^,^^42^.

In this review, we detail the immunological processes underlying the use of RT, as well as the road map for maximum TME reprogramming.

### Unveiling the radiation-induced immune response

Immunogenic cell death (ICD) is characterized by a distinctive response pattern, involving the activation of organellar and cellular stress, ultimately leading to cell death. This process is accompanied by the release of molecules known as damage associated molecular patterns (DAMPs), serving as danger signals that can induce an inflammatory response. DAMPs bind to specific pattern recognition receptors (PRRs) expressed by APC, triggering intracellular cascades that activate both innate and adaptive immune responses. Both, preclinical and clinical data indicate that diverse DAMPs and associated molecules may hold prognostic and predictive value for patients across diverse cancer types.^43^^,^^44^

DAMPs released during radiation-induced ICD include endoplasmic reticulum (ER) chaperones such as calreticulin (CALR),^45^ and heat-shock proteins (HSPs) exposed on the cell surface, the nonhistone chromatin-binding protein high-mobility group box 1 (HMGB1), and the small metabolite adenosine triphosphate (ATP)^46^ released from dying cells.

**Figure 1.**
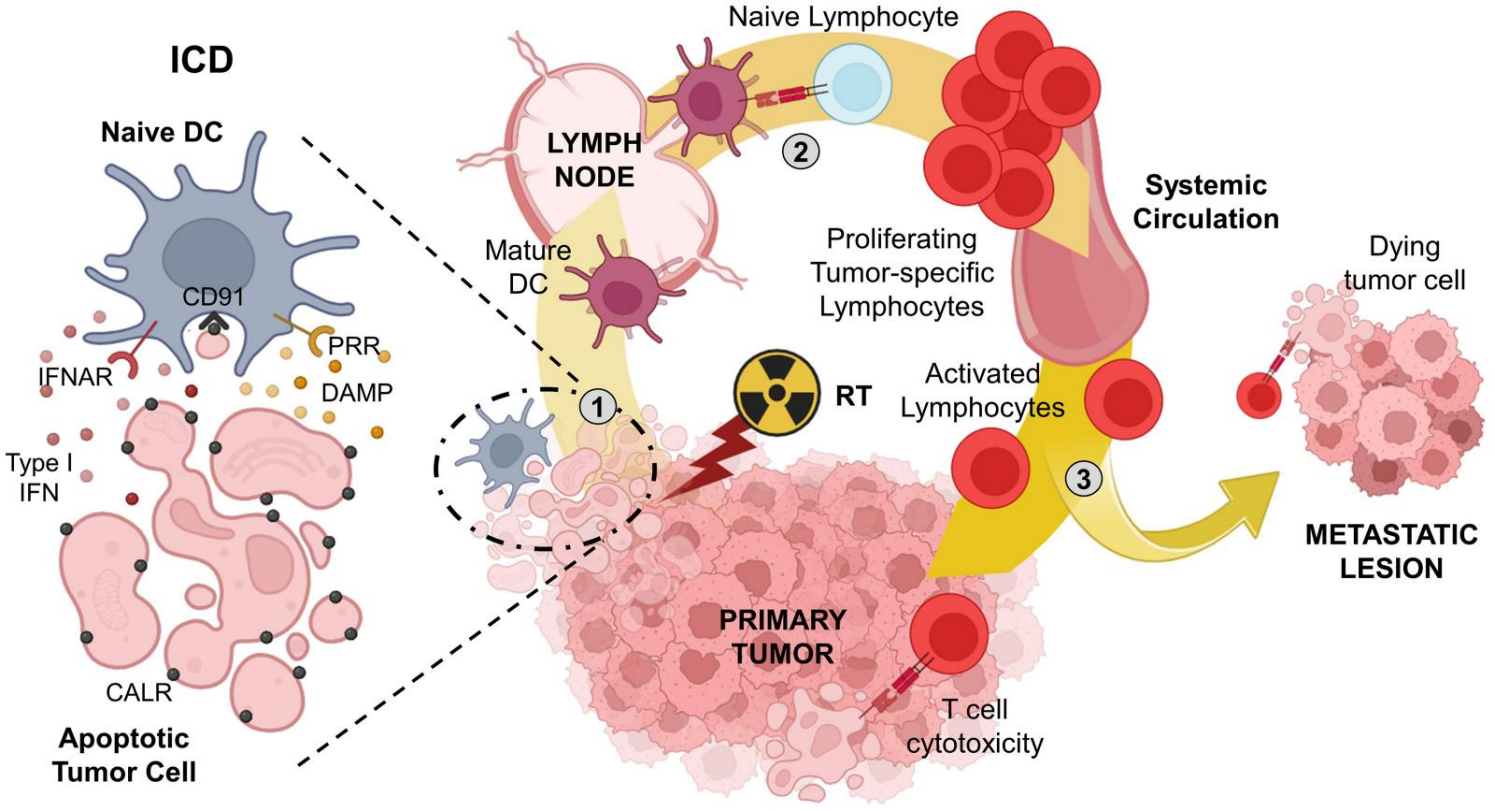
The immunological cell death (ICD) induced by radiotherapy (RT) and it’s *in situ* vaccination effect. (1) The delivery of radiation to the tumour induces the immunogenic cell dead (ICD), characterized by the release of type I interferon (IFN) and cell damage-associated molecular patterns (DAMP). These are recognized respectively by the IFNAR and Pattern Recognition Receptors (PRRs) on the surface of APCs, such as a naïve DC, activating and inducing the maturation of DCs in response to these signals. In addition, the efferocytosis of apoptotic bodies from cancer cells is facilitated by the exposure of calreticulin (CALR) molecules on the apoptotic cell surface. These molecules are recognized by CD91 on APCs, aiding in the acquisition of tumour antigens present in the apoptotic bodies. (2) The activated APCs then migrate to secondary lymphoid organs (SLO), where they encounter naïve tumour-specific T cells, which they activate and promote their expansion. (3) Ultimately, these activated tumour-specific T cells exit the SLO and infiltrate the primary tumour and, if the microenvironment is favourable, also metastatic lesions.

CALR is located on the surface of irradiated tumour cells, where it interacts with CD91 receptors on phagocytes, facilitating the engulfment of apoptotic cancer cells. However, this mechanism is hindered by the presence of CD47 on the surface of cancer cells and signal regulatory protein α (SIRPα) on the surface of macrophages, sending a “*don't-eat-me*” signal to macrophages^47^ (Figure 1).

The release of HMGB1, requires the permeabilization of both the nuclear lamina and the plasma membrane allowing the protein to move from the nucleus to the cytoplasm and then be released into the extracellular environment. HMGB1 binds to receptor of advanced glycation endproducts (RAGE) and toll-like receptor (TLR)2/4, which are expressed in myeloid cells. Phagocytose depends on TLR4 signaling via myeloid differentiation primary response gene 88 (MYD88) on APCs. Agonistic TLR antibodies enhance RT-induced immune responses, contributing to disease control.^43^

ATP secretion through pannexin channels leads to autophagy-dependent ATP release.^48^ Extracellular ATP serves as a *“find-me”* signal for APCs, enabling the recruitment of myeloid cells to active ICD sites through the counterpart receptor P2RX7.^49^

Upon activation of the caspase-1 (CASP1)-dependent nod-like receptor family pyrin domain containing 3 (NLRP3) inflammasome and subsequent secretion of mature IL1β and IL-18, ATP plays a crucial role in mediating pro-inflammatory effects that culminate in T cell activation.^50^ The immunogenicity of cell death diminishes if ATP fails to accumulate in the TME or if P2RX7 or P2RY2 are absent from the myeloid compartment of the host. In tumour tissues, CD73 mediated-degradation of ATP into AMP replenishes the TME with immunosuppressive adenosine, which inhibits immune responses by binding to A2A and A2B adenosine receptors located on immune cells, resulting in a tolerogenic phenotype of DCs, decreased cytotoxic activity of CD8^+^ T cells, and an increase in immunosuppressive macrophages and regulatory Tregs.^51^ Similarly, terminally exhausted T cells express CD39, another enzyme that converts ATP into extracellular adenosine, contributing to the immune-suppressive TME.^52^ CD73 suppression decreases RT-induced Treg infiltration while increasing cDC1 and activating CD8^+^ T cells in the TME, resulting in a more robust immune-response of the irradiated tumour.^51^^,^^53^.

Following RT-induced ICD, a robust type I IFN response ensues, primarily initiated by cytosolic DNA fragments activating cyclic GMP-AMP synthase (cGAS) and its signal transducer stimulator of IFN response cGAMP interactor 1 (STING1).^54^ The cGAS-STING signalling is also involved in promoting a senescent inflammatory cellular response after radiation exposure.^55^ Upon binding to receptors in immune cells, Type I IFN exhibits potent immunostimulatory effects, stimulating APCs, enhancing cross-priming,^36^^,^^56^ inducing cytolytic T cells,^57^ and facilitating immune cell-cancer interactions through the production of tumour-CXCL10 and CCL5.^58^^,^^59^

In addition, RT has the capacity to generate *de novo* neoantigens^60^ and enhance the expression of preexisting ones by upregulating genes involved in cell stress, response to DNA damage, and repair.^32^^,^^61^ This upregulation expands the antigen repertoire expressed by the tumour, promoting an antigen spreading effect. Consequently, this increases the likelihood of tumour cells being recognized by tumour specific lymphocyte. Evidence supporting this phenomenon includes the rapid expansion of selected T cell receptor clones recognizing a neoantigen expressed by the Karyopherin Subunit Alpha 2 (KPNA2) gene, upregulated by RT, in the blood of a nonsmall-cell lung cancer (NSCLC) patient responding to RT and CTLA-4 blockade.^32^ Similar results have been observed in a murine breast cancer model.^62^

Beyond ICD, RT can sensitize tumour cells to T cell recognition. RT has been shown to induce the upregulation of cell surface molecules directly and indirectly, including major histocompatibility complex (MHC)-class I molecules and coactivation molecules, enhancing antigen presentation and lymphocyte activation.^30^^,^^63^^,^^64^ Moreover, RT can induce the expression of death receptors on tumour cells, such as Fas, tumour necrosis factor α (TNF-α),^65^ and TNF-related apoptosis-inducing ligand (TRAIL).^66^ Activated cytotoxic T lymphocytes express ligands for these receptors, and their engagement leads to tumour cell death.^66^ Similarly, radiation upregulates the natural killer group 2D (NKG2D) ligand RAE-1, enhancing NK cell-mediated cytotoxicity.^67^ Collectively, these mechanisms render tumour cells susceptible to recognition by both NK and T cells, contributing to the development of an antitumour immune response. The understanding that radiation operates not only by directly killing tumour cells but also as an immunological adjuvant has generated significant interest in the field, integrating RT into the toolbox of immune-oncology treatments. All these phenomena, along with others induced by RT in the TME, have been comprehensively reviewed elsewhere.^68^

### Challenges and limitations of high dose radiotherapy in clinical practice

Efforts to combine immunotherapy and RT have predominantly focused on high-dose radiation (HDRT), also called hypofractionated RT or stereotactic body radiation therapy (SBRT), which is typically delivered to a few tumour lesions, in a few fractions of >3 Gy, with the aim of inducing diffuse abscopal effects. However, the realization of abscopal effects in combination with ICI has proven elusive in clinical trials.^69–73^

A phase-II clinical trial in head and neck squamous cell carcinoma (HNSCC) patients using nivolumab (anti-PD-1) with SBRT delivered in three fractions of 9 Gy to a single tumour lesion failed to improve overall survival (OS) or progression free survival (PFS), suggesting that the abscopal effect, if present, may be relatively rare.^74^ Similar results were observed in another phase-II clinical trial in patients with advanced NSCLC.^72^ The limitation may arise from irradiating a single metastatic lesion, restricting the antitumour immune response to tumour cells and metastatic lesions sharing the same antigens. As a result, irradiating several metastatic lesions might possibly elicit ICD at each irradiated metastatic site, simulating “multi-site” *in situ* vaccination and increasing the antitumour T cell receptor (TCR) repertoire.^75^ Ollivier et al assessed the occurrence of abscopal effects in 118 metastatic melanoma patients undergoing palliative RT. The abscopal effect ocurred in 12% of the patients. They found that patients experiencing an abscopal response were more frequently treated with immunotherapy (93.3% vs 55.9%, *P* = 0.02) and had a greater number of irradiated metastases treated simultaneously (HR = 16.9, *P* < 0.01).^76^ Multi-site irradiation may necessitate the incorporation of complementary immunotherapy approaches, including CD40 and TLR agonists, as well as ICI, to enhance the effectiveness of HDRT *in situ* vaccination.^29^ Rudqvist et al^38^ showed in a resistant triple-negative murine breast cancer model (4T1) that exclusively blocking CTLA-4 in combination with 3 × 8 Gy RT failed to improve the antitumour efficacy. The addition of PD1 blockade was also ineffective. However, the inclusion of an agonistic CD40 antibody, aimed at enhancing the costimulatory capacity of APCs, in the combination resulted in a superior antitumour response dependent on precursor exhausted T cells. This emphasizes the importance of targeting complementary subsets of the immune population.

Implementation of multi-site radiation requires careful consideration of technical constraints. For instance, the separation of tumour volumes with different isocenters can lead to increased radiation scatter doses between volumes which depend, among other parameters, on the dose and distance between the targets. This, in turn, results in moderate to severe acute toxicities, including haematological toxicity. Notably, in a phase I clinical trial, patients undergoing palliative radiation to the spine, lung, mediastinum, or chest wall experienced severe lymphopenia. This condition further exacerbated after the administration of ICI contributing to the patients’ unfavourable clinical outcomes.^77^ Due to the association of multi-site SBRT with unexpected treatment-related mortality, caution is advised in its implementation.^78^ To mitigate treatment-related toxicity, Luke et al conducted a phase I/II study employing an innovative strategy of partial tumour irradiation. In this approach, two to four metastases received 3-5 fractions totalling 30-50 Gy, followed by pembrolizumab (200 mg intravenously every 3 weeks). SBRT was prescribed up to a tumour volume of 65 mL, allowing for partial metastatic coverage. No significant differences in local control were observed between complete and partial irradiation. SBRT resulted in increased expression of innate and adaptive immune genes and decreased cell cycle and DNA repair genes in irradiated tumours.^79^ Considering clinical data indicating that high tumour burden correlates with reduced responses to ICIs in patients with metastatic melanoma^80^^,^^81^ and NSCLC, strategies such as partial volume or nontumouricidal doses could potentially advance the field of immunotherapy by simply reducing tumour burden.^82^ Huang et al^13^ specifically demonstrated that the magnitude of treatment-induced reinvigoration of circulating exhausted T cells, measured relative to pretreatment tumour burden, correlated with the clinical response to anti-PD1 therapy.

This metric could serve as a valuable tool for identifying patients who might exhibit unresponsiveness to ICI and could potentially benefit from upfront SBRT. In the same line, Darragh et al highlighted the significance of sparing tumour draining lymph node (tdLN) and optimizing surgical resection when combining SBRT with ICI in a murine HNSCC model. While elective nodal irradiation is intended to eliminate the tumour cell reservoir in tdLN, it concurrently hampers T cell priming, crucial for the proper antitumour immune response. Their findings underscore the importance of tdLN sparing during radiation delivery once systemic immunity has developed, leading to improved outcomes in terms of local, distant, and regional recurrence.^83^

Furthermore, in a phase-II clinical trial involving early-stage NSCLC patients, the combination of SBRT (three fractions of 8 Gy) and durvalumab (anti-PD-1) promoted a notable increase in pathological response rate compared to patients treated with durvalumab monotherapy. This augmented pathological response was positively correlated with a significantly elevated expression of MHC-I genes.^84^ Importantly, the same therapeutic approach demonstrated heightened recognition of tumour neoantigens by CD8^+^ T cells in metastatic NSCLC patients.^32^

Despite the synergistic local effects of HDRT with immunotherapy, the immune-mediated destruction of distant and nonirradiated metastasis is uncommon.

Furthermore, the administration of anti-PD-1 prior to RT led to the suppression of systemic immunity due to increased radiosensitivity and death of CD8^+^ T cells, which were induced into cycling by anti-PD1 therapy.^85^ Therefore, RT should be delivered using technologies that minimize damage or detrimental toxicity to surrounding tissues. Importantly, studies have also demonstrated that the timing of ICI administration relative to RT influences treatment efficacy,^85^^,^^86^ underscoring the critical need to optimize the therapeutic schedule for the establishment of an effective antitumour combinatorial treatment.

### Immune-suppressive effects of HDRT: strategies for enhanced efficacy through combinatorial immunotherapies

Despite the myriad of immunostimulatory properties attributed to RT, a growing body of preclinical data suggests its potential for immunosuppressive effects.^87^ Indeed, ionizing radiation has been shown to induce immunosuppression by modulating various cell types, including Tregs, macrophages and MDSC.

Tregs, crucial for regulating inflammation and maintaining self-tolerance can accumulate in the TME and suppress the activation of effector-T-cells through cytokine secretion,^88–90^ resulting in an immunosuppressive impact in the TME. Several research groups have demonstrated that RT can increase Tregs in the TME, suggesting the potential radio-resistance of these cells.^91^^,^^92^

MDSC represent another subset of immune suppressive cells in the TME, constituting a heterogeneous group of immature myeloid cells that accumulate in tumours, disrupting the local immune response induced by RT,^16^ and contributing to tumour progression by supporting tumour cell survival and metastasis.^93^ MDSCs produce immunosuppressive factors such as arginase 1 (ARG1), inducible nitric oxide synthase (iNOS), tumor growth factor beta (TGFβ), and IL-10.^16^ The impact of RT on MDSC varies depending on the RT doses and time points. For instance, RT can induce MDSC infiltration into the TME 3 days after a dose of 15 Gy (3 Gy x 5),^94^ but it can also lead to their decrease 7-14 days after 30 Gy.^95^ Consequently, RT can also induce both the recruitment and reduction of MDSC. As an illustration, 20 Gy RT in a pancreatic cancer cell line increased CCL2 production,^96^ attracting monocytes expressing the corresponding CCR2 receptor to the irradiated tumour and facilitating tumour progression and angiogenesis.^97^ Combinatorial therapies incorporating antiangiogenic drugs, such as bevacizumab (antivascular endothelial growth factor), have the potential to counteract this effect by promoting the normalization of tumour vasculature.^98^ In addition, they can facilitate the generation of tertiary lymphoid structure, potentiating antitumour immune responses.^99^ Furthermore, RT-induced CCL2 production has been linked to the migration of MDSC to MC38 mouse colorectal cancers, resulting in T cell suppression and compromised tumour responses to HDRT.^97^

TAMs, a distinct group of infiltrating immune cells in the TME, exhibit varying roles based on their polarization ranging from pro-inflammatory and antitumoural (M1 polarization) to antiinflammatory and pro-tumoural (M2 polarization). M2 TAMs exert their immunosuppressive actions through the production of antiinflammatory cytokines such as IL-10, TGFβ, and ARG-1, leading to T cell suppression and diminished antitumour immune responses.^100^ In addition, M2 macrophages contribute to angiogenesis, tumour growth and metastasis following irradiation^100–102^ with their presence correlating with decreased patient’s survival. Following RT, TAMs upregulate the phagocytic receptor, MER tyrosine kinase (MerTK). Apoptotic cancer cells induced by RT expose on their outer plasma membrane phosphatidylserine, which coupled with Gas6, interacts with MerTK, promoting the phagocytosis of apoptotic bodies (efferocytosis). Simultaneously, SOCS1 and three expression inhibits TLR and cytokine signalling, further polarizing TAMs towards an M2 profile^103–105^ and facilitating the immune-silent tumour debris clearance.^106^^,^^107^ Moreover, Zhou et al demonstrated that inhibiting MerTK signalling hindered apoptotic tumour cell clearance, promoting secondary cell death that increased cGAS generation. This, in turn, transactivated STING signalling in TAMs, stimulating type-I IFN secretion and inducing TME reprogramming.^108^

Radiation can stimulate tumour-cell production of macrophage-stimulating colony factor 1 (CSF-1), leading to an increased influx of TAMs into the TME, thereby suppressing immune responses.^109^ Furthermore, RT recruits macrophages in mouse models, limiting its efficacy.^110^ Akari et al observed a short therapeutic response after five fractions of 2 Gy in a glioblastoma tumour model. The comparison of responders vs progressors unveiled dynamic and longitudinal changes in the myeloid cell population, including a shift in the ratio of brain-resident microglia to peripherally recruited monocyte-derived macrophages, each exhibiting a distinct gene signature. Targeting TAM populations with a CSF-1R inhibitor alongside RT significantly improved OS.^111^ Hence, a more comprehensive understanding of the impact of ionizing radiation on TAMs is imperative for mitigating local immune suppression and enhancing the immunogenicity of RT.

In a mouse model of rectal cancer (CRC), neoadjuvant radiation therapy (5 fractions of 2 Gy) triggered an elevation of tumour-derived interleukin 1α (IL-1α), leading to the polarization of cancer-associated fibroblasts (CAFs) towards a pro-inflammatory phenotype. The radiation-induced DNA damage in these cells activated a p53-dependent senescence mechanism, resulting in an increased release of extracellular matrix (ECM) components such as collagens. This pathway was associated with CD8^+^ T cell exclusion, contributing to heightened treatment resistance in both mice and patients. Notably, patients with a single-nucleotide polymorphism (SNP) in the IL-1RA receptor antagonist failed to respond to neoadjuvant RT.^112^

Deng et al demonstrated that PD-L1 expression increased on tumour and DCs three days post 12 Gy irradiation, while PD1 expression on CD8+ T cells decreased. This suggests a potential mechanism of radiation resistance that could be addressed by blocking the PD-1/PD-L1 axis.^113^ Notably, Dovedi et al reported that 10 Gy in 5 fractions upregulated PD-L1 on tumour cells,^85^^,^^86^^,^^113^ with maximal upregulation observed at day 3 but reduced by half by day 7^114^; indicating that the window of opportunity for combining RT and ICI is short. In these studies concurrent administration of anti-PD1 or anti-PD-L1 with RT increased OS in murine models.^85^^,^^86^^,^^113^^,^^114^ In patients with oesophageal carcinoma treated with neoadjuvant cisplatin-based chemoradiation, TIGIT, another checkpoint which inhibits costimulatory signals, was upregulated on CD8+, CD4+, and NK cells within four weeks of chemoradiation initiation. To assess whether this effect was mediated by RT, MC38 tumour-bearing mice were irradiated with 15 Gy and sacrificed ten days after, showing increased expression of TIGIT in CD8+ T cells, CD4+ T cells, and NK cells in tumours and tumour-draining lymph nodes compared to expression levels in the same cell populations of nonirradiated control tumours. Radiation improved mouse survival when combined with anti-TIGIT antibodies, either alone with a single 15 Gy dose.^115^ Similarly, 24 Gy in 3 fractions combined with anti-TIGIT and anti-PD-L1 improved antitumour responses in CT26 tumour bearing mice.^116^

An accumulating body of evidence underscores the pivotal role of TGF-β in fostering radiation resistance and anti-PDL1 resistance, creating a peritumoural ECM-rich stroma that excludes T cells from the tumour intra-epithelial compartment.^21^

The combination of radiation with dual targeting of PDL1 and TGF-β has shown superior efficacy compared to monotherapies in cold nonimmunogenic malignancies.^117^ Bintrafusp alfa (BA), a bifunctional fusion protein comprising the extracellular domain of the TGF-RII receptor linked to a human immunoglobulin G1 (IgG1) antibody suppressing PD-L1, demonstrated significant TME remodeling when combined with RT. Bintrafusp, notably decreased fibrosis, ECM, epithelial-to-mesenchymal transition (EMT), angiogenesis, and hypoxia signature scores.^118^ In comparison to RT alone or bintrafusp alone, all these markers were dramatically diminished. In addition, it has been demonstrated that tumour-stroma-produced ECM contributes significantly to RT-resistance. This occurs not only by actively promoting cancer cell survival through integrin signalling (β1 integrin),^119^ mitogen secretion (IL-1α and TGFβ), and oxygen regulation but also by suppressing RT-induced IFN signalling through focal adhesion kinase (FAK) signalling.^120^

Another crucial target within the TME following HDRT is CD47. CD47 acts as a ligand for SIRPα, a protein expressed on macrophages and DCs. Serving as a “*don't eat me*” signal for phagocytic cells, CD47 is expressed on the surface of all human solid tumour cells. Inhibiting CD47 not only amplifies the local antitumour effects of a 10 Gy single-fraction radiotherapy but also triggers abscopal effects in KP1 small cell lung cancer and MC38 colorectal cancer models. The combination of PD-1 and CD47 blockade with 10 Gy irradiation resulted in complete remission of abscopal tumours in KP1 SCLC and MC38 colorectal cancer. This was accompanied by an increase in both macrophages and T cells. The triple therapy was essential for T cell-mediated immune killing.^121^

Finally, radiation-induced immunostimulation is compromised in animals when key components of the type I IFN pathway are artificially removed. Importantly, prolonged type I IFN signalling has an immune suppressive effect on the immune system,^97^^,^^122^^,^^123^ and intrinsic cell specific regulatory mechanism exist. For instance, protein phosphatase 2A (PP2A), with its specific B regulatory subunit Striatin 4 (STRN4), negatively regulates STING-Type I IFN in macrophages.^124^

Cancer cell-derived DNA activates tumour-resident DCs, inducing the production of interferon type I through the cGAS/STING pathway. This process is accelerated in irradiated tumours. Overexpression of the exonuclease TREX1, responsible for degrading double-stranded DNA fragments, inhibits IFN-I production in DCs.^79^^,^^95^^,^^125^ However, chronic IFN-I signalling can have detrimental effects, leading to T cell exhaustion and increased survival of tumour cells.^126^

Therefore, enhancing T cell priming and killing capacity, along with addressing immune suppression in the TME, is crucial for the effectiveness of HDRT in combination with immunotherapy.

### The impact of intestinal microbiota on immune responses following HDRT

Recent findings have linked the microbiota to responses to RT. Shiao et al [127] demonstrated that antibiotic-induced dysbiosis diminishes the tumouricidal effect of 16 Gy RT in breast cancer and melanoma mouse models by fostering the proliferation of intestinal fungi. The administration of antifungal (AF) fluconazole to specific pathogen-free (SPF) mice restored the tumouricidal ability of RT. Immune population analysis indicated that AF treatment combined with RT significantly reduced CD8^+^PD-1^+^ and CD4^+^PD-1^+^ T cells, as well as immunosuppressive CD206^+^F4/80^+^ macrophages, while increasing GzmB-expressing CD8^+^ T cells. Conversely, antibiotic treatment increased the frequency of CD206^+^F4/80^+^ immune suppressive macrophages. Mechanistic analysis, involving depletion experiments and *Candida albicans* inoculations confirmed that the improved efficacy of AF treatment in combination with RT relies on both T cells and macrophages.^127^ In patients with triple negative breast cancer and melanoma, elevated levels of the C-type lectin receptor Dectin-1 (CLEC7A), recognizing β-glucans on fungal cells and sensing pathogenic and commensal fungi, were associated with poorer survival. Furthermore, the beneficial effect of adding AF treatment to RT was nullified in Dectin-1-deficient mice, whereas the response to radiation alone was heightened. These findings underscore the potential influence of increased signals from the commensal microbiota on the response to RT, suggesting avenues for improving radiation responses through diet or microbioma modulation.

Because bacterial depletion is associated with an increase in fungal populations in the gut, the researchers asked whether the effect of bacterial depletion depends on the rise in fungus. tumour-bearing mice were injected with *C albicans*, the most prevalent Saccharomycetales commensal in humans. Surprisingly, the study revealed that *C albicans* growth depletes CD8^+^ T cells, diminishing the antitumour immune-response to RT and reducing OS. Fluconazole, a commonly used antifungal that reduces *C albicans* burden, counteracts the impact, enhancing RT responses and survival while significantly reducing exhausted CD8^+^ T cells. In another study, Teng et al^128^ demonstrated that *Bacteroides vulgatus* increased radioresistance and resistance to five fluorouracil chemotherapy by increasing the production and release of nucleotides, facilitating DNA repair and inhibiting cancer cell death.

Therefore, enhancing T cell priming and killing capacity, along with addressing immune suppression, is crucial for the effectiveness of radiation therapy in combination with immunotherapy.

## The use of low dose irradiation to reprogram the tumour microenvironment

**Figure 2.**
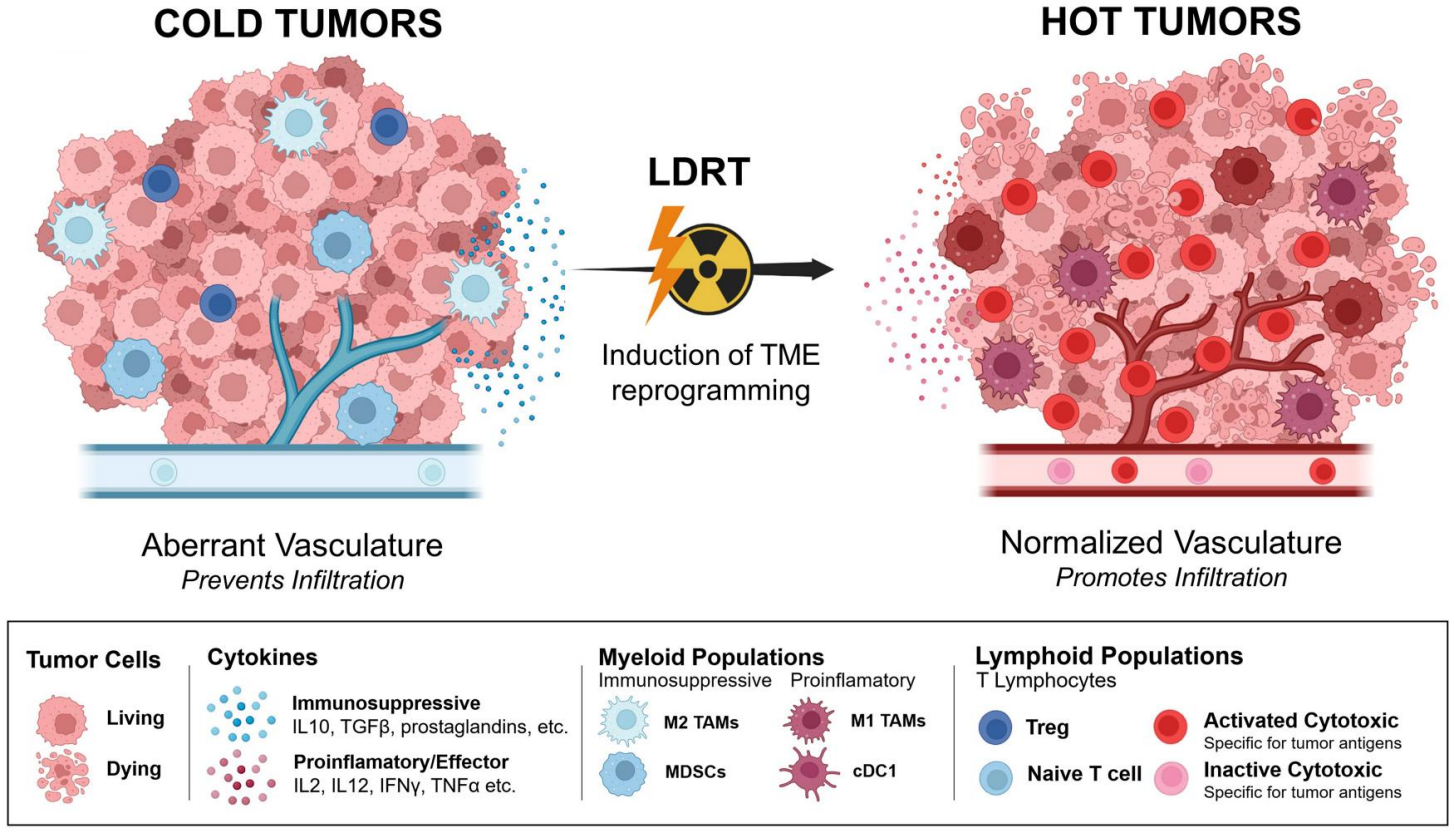
This schematic illustrates the transformative impact of low-dose radiation (LDRT) on converting “cold” tumours into “hot” tumours. Cold tumours, marked by limited infiltration of effector T cells and a prevalence of immunosuppressive myeloid cell populations (such as M2-like TAM) and immunosuppressive cytokines (such as IL-10 and TGF-β), contrast with hot tumours characterized by a robust presence of effector T cells, proinflammatory myeloid cells (such as M1-like TAM or conventional type 1 cDC1), and elevated levels of cytokines (including IL-2, IL-12, IFNγ, TNFα, etc.) among other characteristics.

There is increasing evidence indicating that low-dose radiation therapy (LDRT), specifically doses below the 2 Gy threshold required to induce significant DNA damage or tumouricidal effects, can effectively induce a significant reprogramming of the TME. This transformation turns cold tumours hot, enhancing their susceptibility to ICI therapy as well as adoptive T cell therapy. LDRT can be safely administered to all tumour metastases and to large abdominal volumes in cases of ovarian or gastrointestinal cancers that extensively spread in the peritoneal cavity^129–134^ (Figure 2).

The primary objective of LDRT is to stimulate immune cell infiltration into tumour tissue. In contrast to HDRT, LDRT induces acute stress in tumour cells and minimal DNA damage without reducing tumour growth.^37^^,^^135^

*In vitro* studies revealed that LDRT activates immune cells. Human primary monocytes irradiated with 0.05-0.1 Gy initiate pathways associated with inflammation and survival of cells.^136^ Likewise, murine DCs irradiated with 0.05 Gy produced IL-2, IL-12, and IFN-γ, enhancing T cell activation.^137^ LDRT also has a direct impact on T cells, promoting their proliferation and antigen-dependent cytokine production.^138^ Cheda et al^139^ showed that mice receiving LDRT before tumour inoculation exhibited less lung metastasis, with this effect being immune cell mediated and dependent on the presence of NK cells. These findings collectively highlight the potential of LDRT to directly enhance immune cells.

*In vivo,* the initial evidence of LDRT's contribution to tumour control emerged from preclinical studies, demonstrating enhanced control of metastasis with total body LDRT.^140–142^

Klug and colleagues were pioneers in demonstrating that, in a preclinical model of pancreatic cancer, a single dose of LDRT (0.5-2 Gy) could reprogram immunosuppressive and pro-tumoural M2 TAMs into an antitumoural M1 phenotype characterized by elevated expression of inducible nitric oxide synthase (iNOS). This resulted in the normalization of tumour vasculature, facilitating T cell homing and triggering antitumoural T cell response upon adoptive transfer of autologous lymphocytes.^34^ These findings were confirmed in a clinical study (CT01027221) of advanced pancreatic adenocarcinoma patients, in which local LDRT promoted intraepithelial accumulation of T cells and M1 macrophages.^34^

In another preclinical study, Patel et al presented compelling evidence indicating that the application of LDRT to all tumour deposits through targeted radionuclide therapy (TRT) sensitizes cold tumours to ICIs.^143–146^ Utilizing radionuclide offers the advantage of delivering radiation specifically to all metastatic deposits, including micrometastasis that may not be detectable by imagining.^146^ The investigators administered low-dose TRT, equivalent to 2.5 Gy, in combination with ICIs to immunologically cold syngenic B78 melanoma tumours that exhibit limited response to ICIs alone.^143^ The application of low-dose TRT with ICIs, augmented the immune mediated antitumour response, prolonged OS, and reduced incidence of metastases.

In addition, low-dose TRT monotherapy induced significant TME reprogramming, characterized by elevated infiltration of CD11b^+^ myeloid cells, NK cells, and an increased ratio of effector CD8^+^ T cells to regulatory T cells. TRT treatment also upregulated type I IFN signature, and this effect was dependent on cGAS-STING.^143^ When low-dose TRT was combined with an anti-CTLA-4 antibody, a notable increase in pro-inflammatory cytokine production and expansion of CD8^+^ T cells clonotypes with a less exhausted phenotype were observed compared to monotherapy. Moreover, there was an increase of tumour-resident memory T cells and γδ-T cells.^143^ In the same study, the inclusion of conventional HDRT (12 Gy) targeted at a single tumour significantly heightened the therapeutic effectiveness of low-dose TRT when delivered in conjunction with CTLA-4. Notably, this treatment enhanced the immune response in tumour lesions that were not directly exposed to conventional radiation. Importantly, mice that achieved tumour clearance through the combination developed tumour-specific immune memory.^143^

In a similar way, Barsoumian et al^147^ devised a combined radio-immunotherapy approach in a mouse model of lung adenocarcinoma, involving HDRT (3 × 12 Gy) directed at a primary lesion, LDRT (2 × 1 Gy) for secondary metastases, and immunotherapy (anti-CTLA-4 and anti-PD-1). LDRT induced the repolarization of M2 macrophages into inflammatory M1-like cells and downregulated TGF-β expression in the metastatic lesion. The synergy of HDRT and immunotherapy led to a significant systemic tumour regression, a response shown to hinge on the infiltration of T and NK cells.^147^

After these discoveries, a phase II clinical trial (NCT02710253) was initiated for patients with metastatic cancer, refractory to ICIs. In this trial, patients were treated with HDRT (20-70 Gy) as a standalone treatment or in combination with LDRT (1-10 Gy administered in fractions ranging from 0.5 to 2 Gy) targeting several lesions, while continuing ICIs. This combined treatment facilitated the infiltration of CD8^+^, CD4^+^ and NK cells into irradiated lesions, resulting in an increased overall response rate compared to HDRT alone. Despite the reprogramming of the TME, the trial did not show improvements in disease-free survival nor OS, indicating a challenge in generating a systemic immune-response.^148^

In an effort to explore whether LDRT or HDRT, in combination with ICIs, could induce a systemic immune response, Schoenfeld et al^69^ conducted a phase-II clinical study involving patients with nonresponsive NSCLC to anti-PD-1 or anti-PD-L1 therapy. Patients were treated with durvalumab (anti-PD-L1) and tremelimumab (anti-CTLA-4), either alone or in conjunction with RT, delivered either as hyperfractionated-like (0.5 Gy delivered twice per day, repeated for 2 days during each of the first four cycles of therapy—2 Gy/cycle) or as hypofractionated RT at 8 Gy/cycle (total 24 Gy). The study revealed that, although well-tolerated, the treatment failed to improve objective responses, OS, and PFS compared to RT alone. It noteworthy that RT was selectively directed at a limited number of metastases, which may have been insufficient to enhance the response to PD-L1/CTLA-4 blockade.^69^ Furthermore, most patients received RT targeting lung metastases rather than liver metastases,^69^ potentially impacting the effectiveness of ICI. This discrepancy in the context of liver metastases is attributed to the presence of FasL^+^CD11b^+^F4/80^+^ monocyte-derived macrophages, capable of depleting tumour-specific lymphocytes. This observation aligns with preclinical models where these macrophages, indeed, have been shown to be eliminated upon radiation. Convincing evidence underscores the necessity of directing radiation towards liver metastases to ensure a more robust response to immunotherapy.^149^^,^^150^

In another phase-II clinical trial involving patients with advanced microsatellite-stable colorectal cancer, treated with either hyperfractionated LDRT (2 Gy fractions splitted in 0.5 Gy twice daily for 2 days; total dose of 8 Gy) or hypofractionated RT (3 fractions of 8 Gy each; 24 Gy total dose) combined with durvalumab and tremelimumab and irradiating 1 or 2 liver metastasis similar outcomes were observed.^70^ The study did not reveal significant changes in PFS or OS. Despite the absence of improvements in PFS and OS, the authors reported local and systemic immunological changes marked by the expansion of certain T-cell clones in both the TME and bloodstream, particularly pronounced in patients treated with HDRT, and the presence of macrophages exhibiting an M1 phenotype in the LDRT group.^70^

Our research group contributed additional evidence demonstrating that LDRT induces TME reprogramming and enhances responsiveness to immunotherapy.^37^ In this study, we utilized a syngeneic ID8 mouse model of metastatic ovarian cancer, known for being “cold” and unresponsive to immunotherapy, which accurately recapitulates human epithelial ovarian cancer (EOC).^151–153^ In this model, low-dose whole-abdominal irradiation (0.5-2 Gy) increased the intratumoural frequencies of lymphocytes, monocytes, and NK cells, along with the ratio of CD8^+^ T cells to regulatory T cells, thus transforming the tumour from a “cold” to a “hot” phenotype.^37^ While this effect was transient, with immune cell frequencies declining after one week, repeating irradiation weekly proved effective in promoting durability. Nanostring analysis of tumour tissues five days post-LDRT revealed an upregulation of genes encoding PD-1 and CTLA-4 expressed by exhausted T cells, the regulatory T cell marker FOXP3, and the myeloid stimulatory receptor CD40. Based on the upregulation of these molecules, we designed a 3-week course of orthogonal radio-combinatorial immunotherapy called RACIM, incorporating LDRT (1 Gy), low-dose cyclophosphamide to suppress Tregs, anti-PD-1, and anti-CTLA-4 to enhance T cell activity, and CD40 agonist to activate APCs. RACIM therapy in ID8 ovarian tumour-bearing mice resulted in an 83.5% tumour response and a 15% cure rate. Importantly, all components of the treatment were necessary for the survival benefit.^37^ RACIM led to increased infiltration of tumour-specific activated effector CD4^+^ and CD8^+^ T cells expressing IFN-γ, granzyme B, and perforin. The treatment also induced macrophage repolarization towards an antitumoural M1 phenotype. In addition, RACIM induced DC reprogramming, characterized by the appearance of a new conventional DCs type 2 (cDC2) population expressing a high levels of co-stimulation and a population of monocyte-like DCs expressing a high level of Clec10a and NKG2D ligand RAE1. The costimulatory receptor NKG2D was upregulated in a subset of exhausted T cells with effector and proliferative capacity. Blocking experiments confirmed that the NKG2D receptor was crucial for the antitumoural effect of RACIM. Furthermore, preventing T cell migration from the tdLN abrogated RACIM efficacy, emphasizing the necessity of mobilizing effector T cells from the tdLN for a response.^37^

These findings were translated into a phase I clinical trial (NCT03728179) involving patients with cold nonimmune-infiltrated cancers, defined by a cutoff of fewer than five intraepithelial CD8+ T cells per high-power field. Patients received LDRT (0.5 or 1 Gy) to all visible lesions every two weeks paired with orthogonal immunotherapy. The study included immunotherapy treatment-naïve patients with metastatic ovarian, prostate, gallbladder, or colon carcinoma. Patients received low-dose cyclophosphamide, LDRT, ipilimumab, nivolumab, and aspirin to activate myeloid cells. Similar to murine models, RACIM enhanced patients' tumour immune cell infiltration and regression of solid metastatic disease. Responsive tumours exhibited increased CD4^+^ T cells, Th1, CD8^+^ T cells, and effector memory T cells in tumor islets, whereas nonresponder patients' tumour stroma displayed an increased gene signature of M2-like macrophages. In addition, the treatment reduced tumour size in 37.5% of irradiated metastases. Responder patients experienced regression of all irradiated lesions, with progression observed only in nonirradiated metastases, suggesting a local effect rather than a systemic response. Despite the promising outcomes in preclinical and clinical studies, a significant number of patients who initially responded subsequently progressed, highlighting the need for future investigations into the mechanisms of acquired therapeutic resistance.^37^

## Conclusions and future clinical developments

Understanding the interplay between radiation and immunotherapy is pivotal for advancing combination treatments in clinical practice. However, uncertainties persist regarding the additive, synergistic, or antagonistic effects of these treatments, influenced by factors such as tumour type, treatment sequence, and anatomical sites to be irradiated. Integrating anti-PD1/PD-L1 and SBRT notably improved survival in early-stage NSCLC patients^154^ but yielded disparate results in metastatic NSCLC, underscoring the impact of tumour volume and prior therapies in SBRT and ICI utilization. Moreover, two trials that utilized SBRT before or concurrently with ICI^72^^,^^73^^,^^155^^,^^156^ exhibited a trend towards improved survival, while others that employed ICI before SBRT did not.^69–71^^,^^157^ Standard-of-care approaches, like consolidation immunotherapy postchemoradiation, demonstrated invaluable insights in locally advanced NSCLC^158^ and cervical cancer studies.^159^ However, trials where ICI, chemotherapy and radiation were administered concurrently accompanied by ICI maintenance in unresectable stage III NSCLC have failed.^160^ This interplay of interactions and sequencing underscores that radiation is immunostimulatory, albeit with a narrow window of opportunity.^86^^,^^114^ Radiation activates the immune system, but this effect is counteracted by immune suppression. Hence, implementing strategies such as sparing treatment to TdLN, applying high doses to small volumes to minimize tumour burden and mitigate lymphopenia, as well as administering lower doses of RT to most metastatic deposits for immune reprogramming of the TME may enhance the immune system's response against the tumour when combined with ICI.

In addition, a phase I clinical trial in NSCLC patients revealed superior outcomes with concurrent rather than sequential ablative RT and ICI, particularly in immunologically cold, highly aneuploid tumours.^161^ Notably, RT alone decreased intratumoural cytotoxic T cell and adaptive immune signatures, while RT combined with ICI upregulated key immune pathways.^161^ Hence, biomarker discovery efforts are imperative for predicting treatment responses and optimizing strategies.

The aforementioned observations underscore the need for a profound reevaluation of radiation oncology as a distinct specialty. In the realm of immunotherapy, it invites us to move away from the traditional tumouricidal paradigm and embrace a personalized approach to RT. This approach should take into account not only tumour biology but also the intricate immune landscape and biomarkers, serving as the guiding forces for decision-making. As the field of immunology continues to explore new drug combinations such as STING agonists, CD40 agonists, and TLR agonists with ICI, it becomes increasingly imperative to incorporate these concepts into our approach if we aim to explore these drugs in combination with radiation therapy.^37^^,^^38^

Last but not least, new radiation techniques like LATTICE RT, a leading Spatially Fractionated Radiation (SFRT) method, which delivers heterogeneous radiation doses to tumours, aiming to create higher peaks interspersed with lower dose subregions while sparing surrounding tissues shows promise in clinical settings, especially for nonoperable cancers.^162–165^ Preclinical studies suggest LATTICE RT can trigger immune responses, aiding tumour control and inducing abscopal effects.^166^^,^^167^ However, human immune studies implementing LATTICE RT require further investigation and our group has initiated an immune monitoring assay in metastatic cancer patients to answer to this question.
